# *Halamphora* sp. Reduces Inflammation in LPS-Stimulated Human Malignant Melanoma and Immortalized Keratinocytes Influencing TNF-*α* Release

**DOI:** 10.3390/md24030104

**Published:** 2026-03-10

**Authors:** Eleonora Montuori, Espen Holst Hansen, Calum J. McMullen, Katja Rietdorf, Carlos Almeida, Antera Martel Quintana, Assunta Saide, Chiara Lauritano

**Affiliations:** 1 Ecosustainable Marine Biotechnology, Stazione Zoologica Anton Dohrn, Via F. Acton 55, 80133 Napoli, Italy; eleonora.montuori@szn.it (E.M.); assuntasaide@gmail.com (A.S.); 2 Marbio, Norwegian College of Fishery Sciences, Faculty of Biosciences, Fisheries and Economics, UiT The Arctic University of Norway, N-9037 Tromsø, Norway; espen.hansen@uit.no; 3 School of Life, Health and Chemical Sciences, STEM Faculty, The Open University, Walton Hall, Milton Keynes MK7 6AA, UK; calum.mcmullen@open.ac.uk (C.J.M.); katja.rietdorf@open.ac.uk (K.R.); 4 Banco Español de Algas, Fundación Canaria Parque Científico-Tecnológico de la ULPGC, Telde, 35214 Las Palmas, Canary Islands, Spain; carlos.almeida@ulpgc.es; 5 Banco Español de Algas, Instituto de Oceanografía y Cambio Global, IOCAG, Universidad de Las Palmas de Gran Canaria, Telde, 35214 Las Palmas, Canary Islands, Spain; antera.martel@ulpgc.es

**Keywords:** malignant melanoma, anti-inflammatory, *IL-6/STAT3* signaling, TNF-*α*, marine diatom

## Abstract

Malignant melanoma is skin cancer arising from genetically altered melanocytes. Recently, a complex relationship between melanoma and chronic inflammation has been highlighted, representing an excellent condition for tumor development. Microalgae have been shown to be a promising source of bioactive compounds for drug discovery. In this study, we investigated *Halamphora* sp. (BEA0050) to identify possible compounds with immunomodulatory activity. The most active fraction (fraction D) showed anti-inflammatory activity against human melanoma cancer cells (A2058) stimulated using lipopolysaccharide (LPS) to induce an inflammatory phenotype. Chemical profiling of the bioactive fraction using chromatography and high-resolution mass spectrometry (UHPLC-HR-MS) revealed hydroxypheophorbide *a*, a breakdown product of chlorophyll a. In order to investigate the mechanism of action, the TNF-*α* release was detected through ELISA sandwich assays in A2058 cells and through confocal microscopy in LPS-stimulated HaCaT cells. Gene expression of principal pro-inflammatory cytokines and pathways was detected through real-time PCR, which showed the down-regulation of the inflammatory pathway in LPS-induced A2058 and HaCaT cells treated with 12.5 µg/mL of fraction D. This study reports for the first time the anti-melanoma and anti-inflammatory activities of *Halamphora* sp., identifying protein mediators and highlighting its biotechnological potential.

## 1. Introduction

Melanoma is a cancer affecting the skin, which is one of the first barriers of the innate immune system. This type of cancer originates from malignant proliferation of genetically altered melanocytes, the cells responsible for producing the protective pigment melanin [1,2]. According to the World Health Organization (WHO), more than 500,000 new cases of melanoma per year are estimated and almost 100,000 deaths per year due to melanoma should be expected worldwide by 2040 [3]. Various factors contribute to the development of melanoma, including genetic predisposition. It has been reported that people with phenotypes like red or fair hair, white skin, and blue eyes are at increased risk of developing melanoma compared with other parts of the population, but the risk is also strongly correlated with the exposure to the sun and UV radiation (https://www.who.int/news-room/questions-and-answers/item/radiation-ultraviolet-(uv)-radiation-and-skin-cancer, accessed on 12 January 2026) [1]. Next-generation sequencing on a large scale has identified several genotypic signatures associated with the development of melanoma, such as the mitogen-activated protein kinase and phosphatidylinositol 3 kinase/protein kinase B (PI3K/AKT) pathways which are frequently mutated, amplified, or deleted in malignant melanoma [4]. In melanoma cells, STAT3 (signal transducer and activator of transcription 3) plays a crucial role in promoting tumor growth and progression, not only by regulating cell growth and apoptosis but also by increasing invasiveness and evading the immune response [5,6]. Other important pathways that have implications in inflammation are the Notch signaling pathway that is involved in regulation of tumor microenvironment and metastasis [7] and the c-Jun N-terminal kinases (JNK) pathway involved in proliferation, apoptosis and metastasis [8].

There is a complex relationship between cancer, particularly melanoma, and inflammation [9,10]. Chronic inflammation creates favorable microenvironments for tumor development, promoting cancer progression in various ways [11]. One of these is that malignant cells express numerous pro-inflammatory mediators which support tumor progression and facilitate cancer cells to evade host immune surveillance [12,13]. Cancer cells can evade immune responses, for example through the expression of numerous inflammatory mediators. One of the new frontiers in cancer treatment focuses on reducing inflammation associated with cancer [12]. Hence, the role of inflammation has drawn considerable attention, and it is now considered to be a key factor in cancer initiation and spread throughout the body [14].

It is important to note that melanoma cells themselves can trigger host responses at local and systemic levels by producing neurohormonal regulators (melanoma originates from the neural crest of melanocytes [15]) with immunosuppressive properties, such as intermediates of melanogenesis and melanin that could enhance resistance to various forms of therapy [2]. Melanoma cells have the capacity to stimulate a variety of neuropeptides and neurohormones, including melanocyte stimulating hormone (MSH) derived from proopiomelanocortin (POMC), adrenocorticotropic hormone (ACTH), and β-endorphin peptides, corticotropin-releasing hormone (CRH), urocortins, enkephalins, thyroid-stimulating hormone (TSH), thyrotropin releasing hormone (TRH), neurotrophins, cytokines with pro- and anti-inflammatory properties, catecholamines, serotonin, and melatonin, as well as steroids like corticosterone and cortisol. These molecules can modulate both the melanoma behavior and the responses at local and systemic levels, influencing overall homeostasis in a manner dependent on the specific regulators released and their levels in the circulation [16]. The ability of the tumor to control its own microenvironment phenotype and the body balance poses significant challenges in the therapeutic fight for patient survival [16].

Currently, various treatments are being used to treat melanoma, ranging from traditional chemotherapy using anti-proliferative drugs to immunotherapy that focuses on inflammatory mediators [17].

The aim of our experiments focused on exploring the potential immune system-modulating effects of the marine diatom *Halamphora* sp. (Bacillariophyta family and Bacillariophyceae class; https://www.ncbi.nlm.nih.gov/Taxonomy/Browser/wwwtax.cgi?id=1951536; accessed on 12 January 2026). Diatoms, which make up 20% of the global primary biomass productivity [18], are a significant source of fatty acids, including omega-3 and omega-6 unsaturated fatty acids, which are crucial for human health [19]. Numerous compounds extracted from microalgae exhibit a multitude of activities in different physiological processes, interacting with different cellular targets [1,20,21,22,23,24,25,26,27,28,29]. *Halamphora* sp. is known to be particularly abundant in eicosapentaenoic acid (EPA) and docosahexaenoic acid (DHA) fatty acids, as recent studies have indicated [30,31]. While there is a growing interest in the potential use of *Halamphora* sp. in aquaculture, its practical application in the pharmaceutical, cosmeceutical, and nutraceutical sectors remains largely unexplored [30]. In the current study, we used a multidisciplinary approach to highlight the activity of *Halamphora* sp. (by following a bioactivity-guided fractionation approach), the chemical composition of the most active fraction, and its mechanism of action related to the anti-inflammatory activity in human cells.

## 2. Results

### 2.1. Chemical Extraction, Fractionation and In Vitro Antiproliferative Activity of Halamphora *sp.*

**Figure 1:**
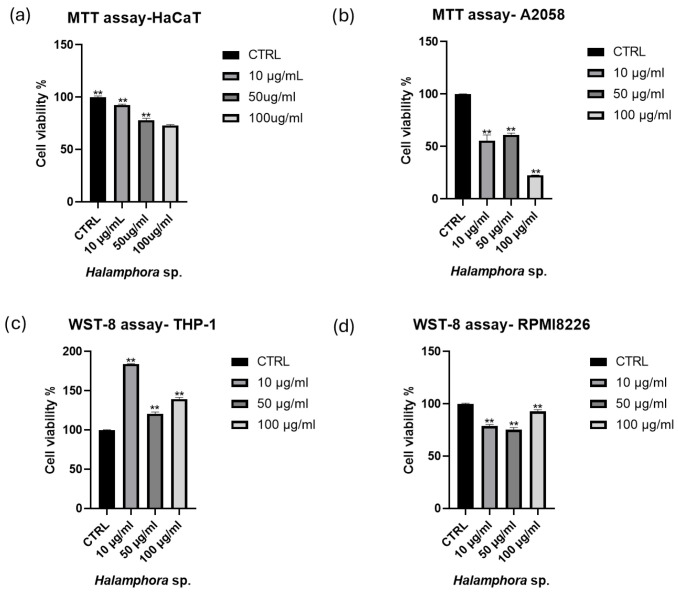
Cell viability assay. The figure shows the effects of raw extracts of *Halamphora* sp. on cell viability at increasing concentrations (10, 50, 100 μg/mL). Effects on (**a**) human normal immortalized keratinocytes HaCaT and (**b**) malignant melanoma A2058 cells were tested by MTT assay. Effects on (**c**) THP-1 and (**d**) RPMI8226 cell lines were tested by WST-8 assay. Cell viability was normalized to control cells (0.5% DMSO addition). Results are expressed as percent survival after 72 h exposure (n = 3; ** for *p* < 0.002, ordinary one-way ANOVA, Dunnett’s post hoc test).

The effect of the *Halamphora* sp. raw extract on the proliferation of different cancer cells and human HaCaT cells, assessed via MTT assay, is reported in Figure 1. We defined the extract as ‘active’ if it reduced cell viability of cancer cell lines below 50%, relative to the control. The raw extract of *Halamphora* sp. reduced cell proliferation in all cell lines tested (Figure 1), but based on our definition of an active sample, the raw extract of *Halamphora* sp. was only active against the A2058 cell line (Figure 1b). In fact, raw extract of *Halamphora* sp. was able to reduce A2058 viability to 22.6 ± 0.29% when tested at 100 μg/mL. A2058 are highly metastatic human malignant melanoma cells and are commonly used to study melanoma cells and to investigate their correlation with inflammatory responses seen in melanomas.

**Figure 2:**
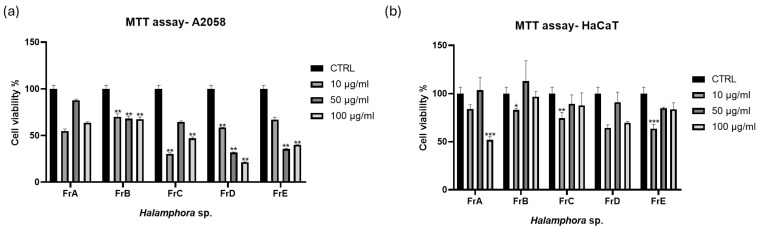
Cell viability assay. The figure shows the effects of Halamphora sp. fractions on cell viability. MTT assay was used to test increasing concentrations (10, 50, 100 μg/mL) of fractions on (**a**) A2058 and (**b**) HaCaT cell lines. Cell viability was normalized using cells with only DMSO (0.5%) as control sample. Results are expressed as percent survival after 72 h exposure (n = 3; * for *p* < 0.033, ** for *p* < 0.002, and *** for *p* < 0.001, two-way ANOVA, Dunnett’s post hoc test).

Using a solid phase extraction (SPE) method with carefully selected elution solvents, we performed the fractionation of the raw extracts, obtaining five fractions enriched in specific classes of molecules. As previously described [32], for fraction A, we used H_2_O to elute amino acids and saccharides; for fraction B, methanol (MeOH)/water (H_2_O) 50:50 to elute nucleosides; for fraction C, acetonitrile (CH_3_CN)/H_2_O 70:30 to obtain glycolipids and phospholipids; for fraction D, CH_3_CN 100% to elute free fatty acids and sterols; for Fraction E, dichloromethane (CH_2_Cl_2_)/CH_3_OH 90:10 to obtain triglycerides [32]. The cytotoxicity of the fractions of *Halamphora* sp. was tested using A2058 cells and the most active fraction was fraction D, with a concentration-dependent activity (Figure 2a). Fraction D reduced viability to about 58.5 ± 0.15% at 10 μg/mL, about 31.9 ± 0.55% at 50 μg/mL, and about 21.8 ± 0.42% at 100 μg/mL, after 72 h treatment. The same fraction D did not show cytotoxicity on HaCaT normal cells (Figure 2b). Fraction C and fraction E also showed an antiproliferative effect on A2058 cells, but less pronounced and without dose-dependent activity.

### 2.2. Metabolomic Profiles and MS of Fraction D

**Figure 3:**
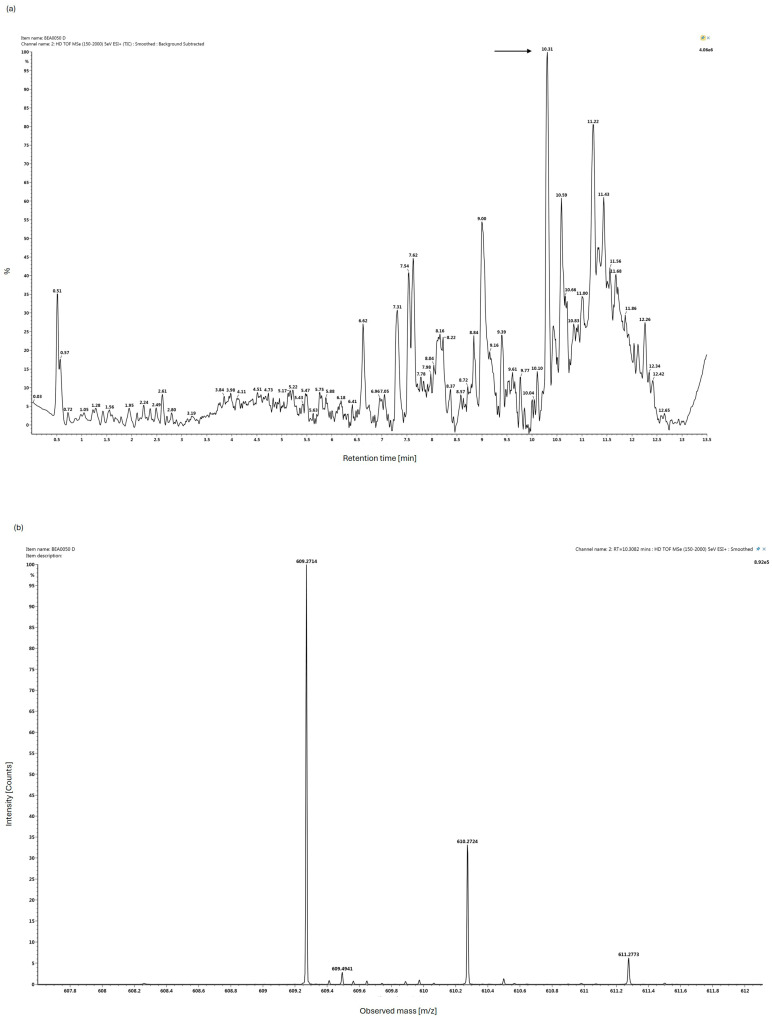
UHPLC-HR-MS/MS analysis of bioactive fraction D reveals hydroxypheophorbide *a* as a major component (The arrow indicates the peak identified as hydroxyphaeophorbide *a*). The upper panel (**a**) shows a background subtracted ion chromatogram in ESI+, and the panel below (**b**) shows the accurate mass spectrum of the peak eluting at Rt 10.31 min.

The bioactive fraction was analyzed using ultra-high performance liquid chromatography and high-resolution tandem mass spectrometry (UHPLC-HR-MS/MS) to dereplicate the compounds responsible for the observed bioactivity. Fraction D was non-polar as it was eluted from the resin with 100% CH_3_CN and this was reflected in the mass chromatogram, as relatively few signals with short retention times could be observed in the background subtracted chromatogram (Figure 3). By comparing the chromatograms from active and inactive fractions, it was possible to identify the peaks that were unique to the bioactive fraction D. The main and most abundant peak that was unique to the bioactive fraction eluted at 10.31 min, and it had an accurate mass of *m*/*z* 609.2714 corresponding to an elemental composition of C_35_H_36_N_4_O_6_ ([M + H]^+^ calculated as 609.2713). The elemental composition and the fragmentation pattern of the compound indicated that it was a breakdown product of chlorophyll, namely hydroxypheophorbide *a*, a compound that is known for having anti-inflammatory effects [33]. Hydroxypheophorbide *a* is a macrocycle consisting of four modified pyrrole rings, but unlike chlorophyll a, the central magnesium ion is removed during the degradation process.

### 2.3. The Effect of Fraction D on A2058 Is Time- and Concentration-Dependent

**Figure 4:**
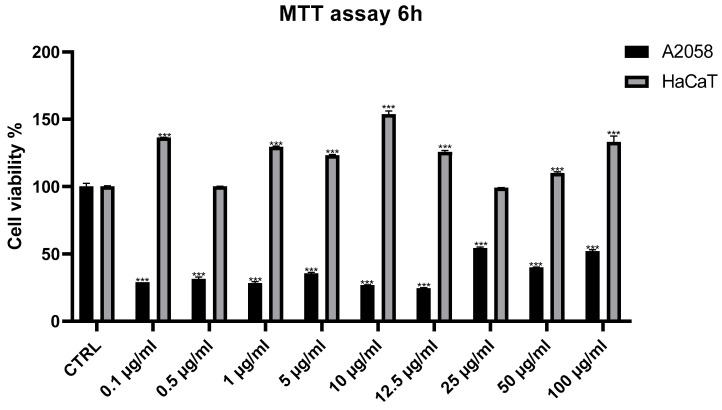
Cell viability assay. The figure shows the effects of *Halamphora* sp. fraction D on cell-viability. MTT assays were used to test increasing concentrations (0.1, 0.5, 1, 5, 10, 12.5, 25, 50, 100 μg/mL) of fraction D on A2058 and HaCaT cell lines. Cell viability was normalized using cells with only DMSO (0.5%) as control sample. Results are expressed as percent survival after 6 h exposure (n = 3; *** for *p* < 0.001, two-way ANOVA, Dunnett’s test).

As fraction D was the most active fraction, it was selected for further experiments. We aimed to investigate whether fraction D exhibited anti-inflammatory activity in malignant melanoma cells, which are frequently used in immunomodulatory studies [34]. This experimental approach was further supported by the correlation between melanoma and chronic inflammation [35]. Fraction D is rich in free fatty acids and sterols, and it is known that these classes of molecules have anti-inflammatory and anticancer properties [36,37]. We increased the range of concentrations (0.1, 0.5, 1, 5, 10, 12.5, 25, 50, 100 μg/mL) and exposure times (6 h, 24 h, 48 h, 72 h) of fraction D, to find the concentration and time at which fraction D started to have activity on melanoma cells. Based on the results from the time- and concentration-dependent antiproliferative experiments, we selected the 12.5 μg/mL concentration. This activity became evident after 6 h of treatment (Figure 4 and Supplementary Figure S1). Interestingly, treatment with fraction D did not reduce the viability of healthy HaCaT cells. On the contrary, a significant increase in cell viability was observed compared to the untreated controls (Figure 4).

### 2.4. Immunomodulatory Activity of Fraction D on A2058

#### 2.4.1. Lipopolysaccharide-Induced Inflammation in A2058

Once we had defined concentration and exposure time for naïve A2058 cells, we were interested in how inflamed A2058 cells respond to fraction D. We exposed A2058 cells to lipopolysaccharide (LPS) to trigger an inflammation response. As demonstrated in the literature [13], LPS induces an increase in migration and invasion in A2058 cells, accompanied by an increase in inflammation products. In this cell line, the pro-inflammatory cytokine interleukin-6 (IL-6), the chemokine interleukin-8 (IL-8) and tumor necrosis factor-α (TNF-*α*) are constitutively expressed [13,38]. We treated the A2058 cells with 1 µg/mL of LPS [13] and with fraction D from *Halamphora* sp., and after 6 h of incubation, we performed a total RNA extraction to investigate which pathways were influenced by fraction D.

#### 2.4.2. Fraction D Reduced Inflammation and Increased Apoptosis at Transcriptional Level

**Figure 5:**
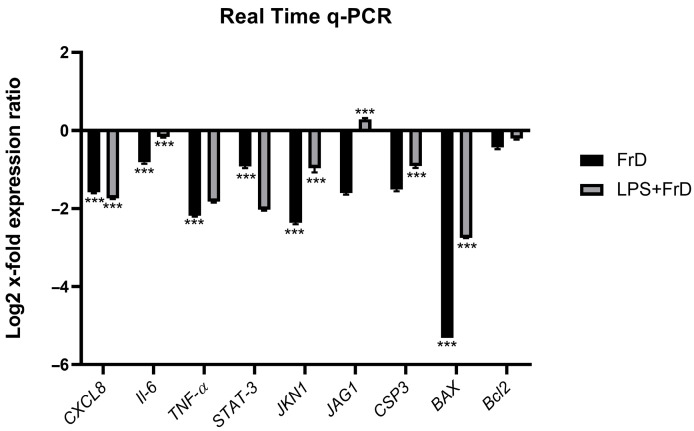
Real-time PCR. Expression levels of genes involved in inflammatory pathways in LPS-induced A2058 cells treated with fraction D. The results are expressed as log2 fold changes. Statistical analyses were performed using two-way ANOVA, Tukey’s test (*** *p*  <  0.001). *RPLP0* was used as reference gene.

We examined different pathways that were triggered during inflammatory responses and tumorigenesis. We considered *STAT3* expression levels to evaluate the influence of fraction D on the *JAK/STAT3* pathway that plays a role in the expression of various critical inflammatory mediators, *jagged 1* (*Jag1*) for the Notch signaling pathway that plays a role in the tumorigenesis and malignant transformation, and *JNK1* for the *JNK* pathway that is involved in metastasis, apoptosis, and proliferation. As shown in Figure 5, fraction D reduced the expression of pro-inflammatory cytokines *IL-8*, *IL-6*, and TNF-*α* in LPS-stimulated A2058 cells. As we expected, *STAT3* was down-regulated. *STAT3* is an oncogene and a transcription factor that has an immunosuppressive role and, as previously demonstrated, when its activation is inhibited, it improves the anticancer immune response [5]. It is known that *IL-6* is responsible for the activation of *STAT3*, and we observed down-regulation of both *IL-6* and *STAT3*. We also found down-regulation of *STAT3* related genes, such as *Jag1*, which is part of *Notch* pathway involved in metastasis [39]. *CXCL8* was down-regulated. This is a gene encoding IL-8, a chemokine with a pro-tumorigenic role promoting tumor survival by the inhibition of anticancer immunity [40]. In particular, IL-8 can recruit tumor-associated macrophages (TAMs), myeloid derived suppressor cells (MDSCs), and neutrophils to the tumor micro-environment (TME) [41]. Normally, the up-regulation of *IL-8* is related to the activation by *JNK* pathway [42]. Fraction D induced a down-regulation of c-Jun N-terminal kinases (*JNK-1*) involved in proliferation, survival, migration, and apoptosis in melanoma [43,44]. In fact, dysregulation of *JNK-1* is associated with a wide range of skin diseases and cancer types. Normally, the *JNK* pathway is activated by external stimuli. One of these is the cytokine TNF-*α*. The *JNK* and *JAK/STAT* pathways are known to act synergistically to promote cell survival [45]. As demonstrated in the literature, when *JKN1* is down-regulated, apoptosis decreases [44,46]. Our results are in line with the down-regulation of *BAX* and Caspase-3 expression levels. In contrast, *Bcl-2*, an anti-apoptotic gene, did not show significant changes in expression level with fraction D treatment.

To assess whether the observed effects were specific to cancer cells, we also treated a non-tumorigenic human keratinocytes HaCaT with fraction D under the same experimental conditions and analyzed expression of the same genes. As shown in the graph provided in the Supplementary Materials (Supplementary Figure S2), after 6 h of treatment with fraction D, we observed a significant down-regulation of pro-inflammatory genes such as *CXCL8*, *IL-6* and TNF-*α*. Moreover, genes associated with signal transduction and oxidative stress were consistently down-regulated both in cells treated with fraction D alone and those previously stimulated with LPS. These findings suggest that fraction D may act on pro-inflammatory and pro-apoptotic signaling pathways. Apoptosis-related genes such as *CASP3* and *BAX* were significantly suppressed following fraction D treatment, indicating a potential cytoprotective effect. Conversely, *Bcl-2*, an anti-apoptotic gene, showed modest up-regulation, further supporting the hypothesis of a protective role of fraction D on epithelial cells (HaCaT cells). Overall, these data demonstrate that the bioactive fraction D exerts both anti-inflammatory and cytoprotective effects on HaCaT cells by reducing the expression of LPS-induced pro-inflammatory and pro-apoptotic genes, as determined by direct statistical comparison with LPS-treated control. These results reinforce the therapeutic potential of the marine microalga as a modulator inflammatory pathway in melanoma cells used as a model system.

#### 2.4.3. Fraction D Reduces TNF-*α* Release in A2058

To investigate the expression of the inflammatory cytokine TNF-*α* at protein levels, we performed an enzyme-linked immunosorbent assay (ELISA). We treated cells with the active concentration (12.5 μg/mL) of fraction D for 6 h and we compared the release of TNF-*α* in A2058 cells stimulated with LPS and not stimulated. A2058 with only media and vehicle were considered as the control.

**Figure 6:**
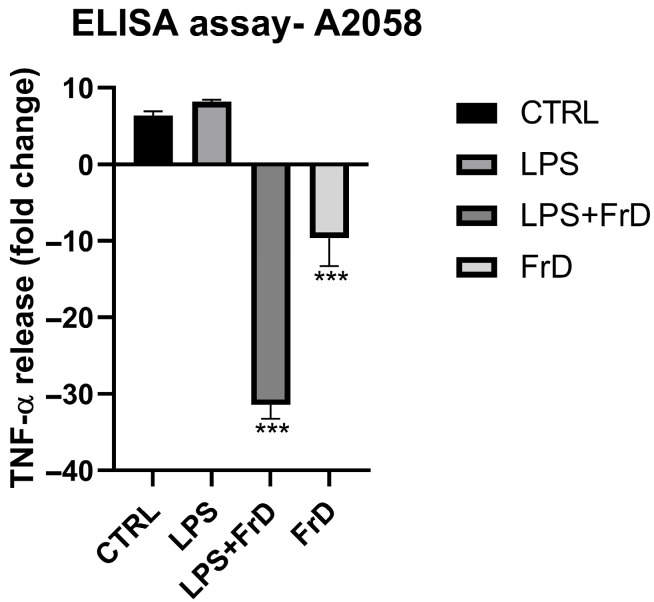
Enzyme-linked immunosorbent assay (ELISA) was used to measure the release of TNF-*α* in A2058 stimulated with LPS and treated with fraction D of *Halamphora* sp. Data were normalized using cells with only DMSO (0.5%) as control sample (n = 3) Statistical analysis was performed using one-way ANOVA followed by Dunnett’s post hoc test. Asterisks indicate statistically significant differences compared to LPS-stimulated cells treated with vehicle (*** for *p* < 0.001).

As reported in Figure 6, the treatment with fraction D induced decreases in A2058 TNF-*α* release in non-stimulated melanoma cells and in LPS-stimulated cells compared to the control conditions, of about −10 and −30 fold, respectively.

### 2.5. Confocal Immunofluorescence Analysis of TNF Alpha in HaCaT LPS Stimulated Cells Treated with Fraction D

**Figure 7:**
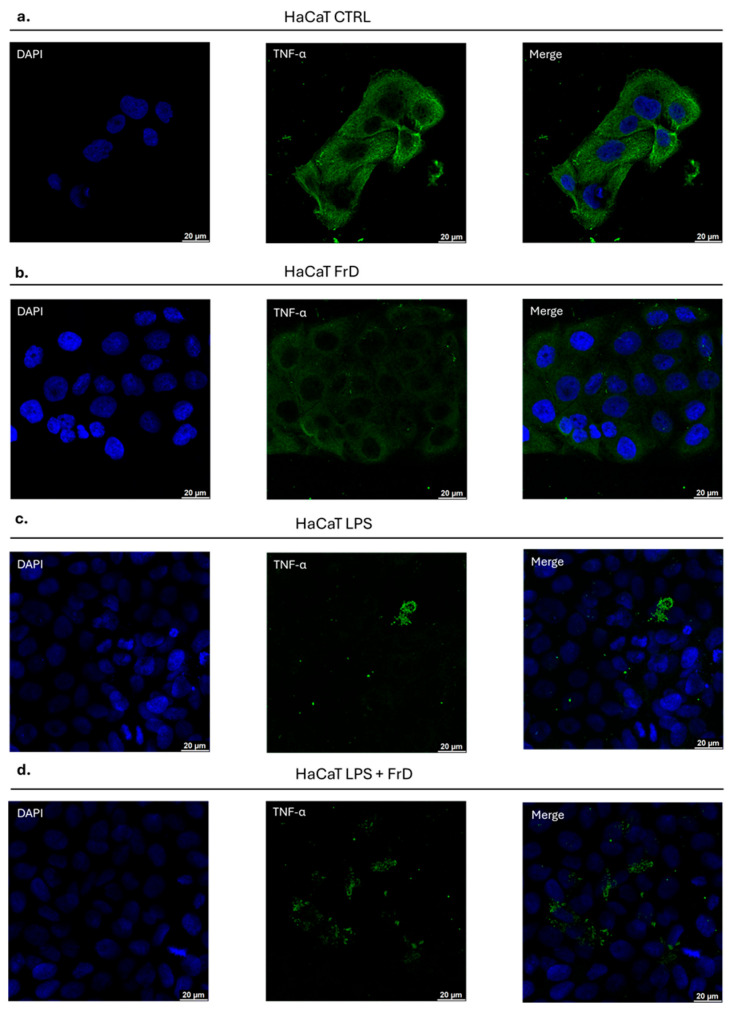
Confocal immunofluorescence analysis of (**a**) HaCaT in control conditions, (**b**) HaCaT treated with FrD (Fraction D), (**c**) HaCaT stimulated with LPS, (**d**) HaCaT stimulated with LPS and co-treated with FrD. The cells were stained with anti-human TNF-*α* (green fluorescence) and DAPI (blue fluorescence). Scale bar 20 µm.

Many reports have documented that upon exposure to an infectious agent, HaCaT keratinocytes are able to produce inflammatory cytokines to activate the immune response against the pathogens [47]. It is well known that LPS induces an inflammatory response in HaCaT cells, which then produce the cytokines TNF-*α* and IL-6 after a period of 6 h [47]. To investigate the possible anti-inflammatory activity of fraction D of *Halamphora* sp., we compared the localization of TNF-*α* in control cells (DMSO treatment) and cells treated for 6 h with either LPS, fraction D, or both. Cells were then fixed and stained for immunofluorescence as described in Section 4. TNF-*α* release was visualized using confocal microscopy, shown in Figure 7. The results showed that TNF-*α* was found inside the control cells (DMSO only, Figure 7a). Similarly, TNF-*α* staining was visible inside the cells after treatment with fraction D only (Figure 7b). In contrast, after 6 h of treatment with LPS only very little TNF-*α* could be observed in the cell, but some was seen on the cell membrane (Figure 7c). The majority of TNF-*α* will have been released from the cells and washed away during processing of the cells. To test the effects of fraction D, HaCaT cells were pre-incubated with LPS for 1 h before addition of the active concentration of fraction D for 6 h. After fraction D treatment, TNF-*α* staining was visible in the cells (Figure 7d), indicating that fraction D reduced the TNF-*α* release and showing that the fraction D was able to block the inflammation induced by LPS.

## 3. Discussion

Over recent decades, numerous studies have highlighted a close correlation between melanoma and the inflammatory processes present in the body. As also demonstrated by the genetic data obtained by Lu et al. in 2024 [9], there is an association between the expression of pro-inflammatory markers, including IL-6, IL-8, TNF-*α*, and the onset of melanoma. Inflammation is considered an important factor in the onset and development of melanoma and tumors in general. It influences both the tumor microenvironment and the immune response mounted by the body against the tumor. Therefore, further research in this direction could be a step towards the clinical application of new approaches targeting inflammatory markers. Understanding this correlation between melanoma and inflammation is of fundamental importance for research into new diagnostic and therapeutic strategies aimed at effectively combating cancers, including skin cancer. In our study, following induction of an inflammatory stimulus with LPS in A2058, we evaluated the possible immunomodulatory activity of the fraction D of an extract of *Halamphora* sp. We analyzed the main pathways activated during the inflammatory response, which are already constitutive expressed in A2058 melanoma cells. We found that fraction D reduced the release of *IL-8*, *IL-6*, and TNF-*α*. TNF-*α* is directly involved in the generation of reactive oxygen species [48] and is a therapeutic target for treatment of chronic inflammatory diseases. Indeed, biologic agents such as infliximab, etanercept, and adalimumab effectively inhibit the action of TNF-*α* and reduce inflammation [49,50].

One of the main chemical constituents in the bioactive fraction D of the *Halamphora* sp. extract was hydroxypheophorbide *a*. This compound, a breakdown product of chlorophyll a, has a porphyrin core structure consisting of four modified pyrrole units. Hydroxypheophorbide *a*, together with other related porphyrin compounds, has previously demonstrated significant anti-cancer, antioxidant, and anti-inflammatory activity [51,52]. These bioactivities highlight its therapeutic potential in cancer and inflammatory conditions. In previous studies, we identified pheophorbide *a* (C_35_H_36_N_4_O_5_ as a protonated species at *m*/*z* 593.275) together with hydroxypheophorbide *a* in bioactive microalgal samples [33]. However, in this sample, we did not detect pheophorbide a.

The mRNA down-regulation of *STAT3* that we observed reflects the down-regulation of *IL-6* mRNA, because *IL-6* during the immune inflammatory response promotes the overexpression of *STAT3* increasing migration, proliferation, and survival in cancer cell [53]. *STAT3* is an oncogenic transcription factor with immunosuppressive function, and its action has also been associated with an increased probability of relapse of melanoma after therapy [5,54]. For this reason, the *IL-6/STAT3* axis could be considered a good target for new cancer immunotherapy. *STAT3* has resistance to the drug vemurafenib, already in use for melanoma. Therefore, research into new compounds with pharmacological value directed towards *STAT3* could support the resolution of the problem [55,56,57].

An important aspect to consider is that melanoma is a disease highly resistant to current radiotherapies and chemotherapy. Moreover, many of the drugs currently used in cancer therapy are highly toxic and have devastating side effects. In addition, the incidence of melanoma increases with age, and a significant proportion of elderly patients are not suitable candidates for surgical intervention, mainly due to the risks associated with anesthesia. For this reason, it is essential to develop new therapies that are less toxic and have fewer side effects [58]. We found that fraction D was toxic to cancer cells but was not toxic to normal cells (it even increased the cell viability), making it a promising product to study for the development of new therapies. Another frontier of cancer therapy is immunotherapy with cytokines that seeks to exert toxic effects directly on cancer cells increasing the soft response of the body [59].

Considering the increasing demand of new drugs, the attention of researchers is focused on natural products. Marine microalgae represent a fascinating resource of natural compounds due to their enormous diversity in terms of species and also to their versatility and adaptability to changing growing conditions, with the possibility of improving the production of products of interest [60].

## 4. Materials and Methods

### 4.1. Microalgae Culturing and Harvesting

Lyophilized biomass of *Halamphora* sp. (BEA 0050) was obtained from the Spanish Bank of Algae (BEA). Microalgae were cultured in Guillard’s medium in quadruplicate using 7-L PET (polyethylene terephthalate) photobioreactors. Microalgae were cultured in a growth chamber at 25 ± 2 °C and continuous aeration supplied with CO_2_ pulses addition at a rate of 1 min every hour. Illumination was provided by cool-white LED tubes (Sysled GmbH, Berlin, Germany) with an intensity of 145 ± 10 μmol photons m^−2^ s^−1^ under a 16:8 h light–dark cycle. Microalgae growth was monitored daily by measuring the fresh weight of the whole biomass retained on a 50 µm mesh filter. Biomass replicates (G1 to G4) were harvested when cultures reached the stationary phase and freeze-dried (6.5 L Labconco, Kansas City, MO, USA) before being weighed and stored in dark screw cap bottles. Purity of the cultures was periodically assessed with a light microscope (Leica DM2000 microscope, Leica Microsystem, Weltzlar, Gemany).

### 4.2. Chemical Extraction

Freeze-dried biological pellets of *Halamphora* sp. BEA0050 were extracted in MeOH (1:5, *w*/*v*). The biomass was sonicated (Sonicator BEDELIN, Berlin, Germany) with three bursts of 30 s in an iced water bath to lyse the cells, before being centrifuged at 3800 rpm at room temperature to precipitate the solid material. The organic phase was transferred to glass vials, decanted and dried under vacuum. The extracts were stored at −20 °C until use. Fractionation of raw extract was performed by solid phase extraction (SPE) using CHROMABOND HR-X cartridges (6 mL/500 mg) (Macherey-Nagel, Düren, Germany) as in Cutignano et al. [32]. The cartridge was conditioned with 3 mL of MeOH and equilibrated with 6 mL of distilled water. The extract was prepared with the addition of 1 mL of distilled water and sonicated for a few seconds in an iced water bath, to promote the mechanical disruption of residual cellular structures, before loading it into the cartridge. After the extract was absorbed by the resin, the elution consisted of five steps to obtain five different fractions: 100% H_2_O (12 mL, fraction A); CH_3_OH/H_2_O (50:50, 18 mL, fraction B); CH_3_CN/H_2_O (70:30, 12 mL, fraction C); 100% CH_3_CN (12 mL, fraction D); CH_2_Cl_2_/CH_3_OH (90:10, 12 mL, fraction E). The fractions, collected in vials, were decanted and dried under vacuum and preserved at −20 °C.

### 4.3. UHPLC-HR-MS Analysis of Bioactive Fractions

UHPLC-HR-MS data for identification of potential bioactive compounds in the fractions was acquired using an Acquity I-class UPLC (Waters, Milford, MA, USA) coupled to a PDA detector and a Vion IMS QToF (Waters). Nitrogen was used for desolvation at 600 L/h and 350 °C, the capillary voltage was 0.8 kV and the sample cone was operated at 30 V. The chromatographic separation was performed using an Acquity BEH C18 UPLC column (1.7 µm, 2.1 mm × 100 mm) (Waters). The mobile phases consisted of H_2_O for mobile phase A and acetonitrile (HiPerSolv, VWR, Radnor, PA, USA) for mobile phase B, both containing 0.1% formic acid (*v*/*v*) (Sigma-Aldrich, St. Louis, MO, USA). The gradient was run from 10% to 90% B over 12 min at a flow rate of 0.45 mL/min. Samples were run in ESI+ ionization mode. The data was processed and analyzed using UNIFI 1.9.4 (Waters).

### 4.4. Cell Culture

Human cells were purchased from ATCC (https://www.atcc.org/) (Manassas, VA, USA). A2058 cells (human melanoma; ATCC-CRL-11147) and HaCaT cells (spontaneously immortalized keratinocytes from adult skin) were cultured in DMEM High Glucose supplemented with 10% fetal bovine serum, 1% L-glutamine and 1X pen–strep solution in a humidified incubator at 37 °C and 5% CO_2_. RMPI 8226 (human multiple myeloma; ATCC-CRM-CCL-155), U937 (human histiocytic lymphoma monocyte; ATCC-CRL-1593.2™) and THP1 (human leukemia monocyte; ATCC-TIB202™) were cultured in RPMI 1640 media supplemented with 10% fetal bovine serum, 1% L-glutamine and 1X pen–strep solution in a humidified incubator at 37 °C and 5% CO_2_.

### 4.5. MTT Assay

To estimate the in vitro antiproliferative effects, HaCaT and A2058 cells were seeded in 96-well microtiter plates at a concentration of 1 × 10^4^ cells/well and incubated at 37 °C in a humidified cell culture incubator. After 24 h, the medium was replaced with fresh medium containing increasing concentrations of the total extract and fractions dissolved in dimethyl sulfoxide (DMSO) and further incubated for 72 h. The maximum concentration of DMSO used was 0.5% (*v*/*v*). Each concentration was tested at least in triplicate. After 72 h, cell viability was assessed using the MTT assay 3-(4,5-dimethyl-2-thiazolyl)-2,5-diphenyl-2H-tetrazolium bromide; A2231,0001, Applichem Panreac, Darmstadt, GmbH, Berlin, Germany). The medium was replaced with medium containing MTT solution 1:10 (stock solution 5 mg/mL) and the plates were incubated for 3 h at 37 °C. After incubation, cells were treated with isopropyl alcohol (used as MTT solvent) for 30 min at room temperature. Absorbance was measured at OD = 570 nm and 630 nm as backgrounds, using a microplate reader (Synergy™ HTX Multi-Mode Microplate Reader, Agilent, Santa Clara, CA, USA). Cell viability was expressed as a percentage of viable cells in the presence of the tested samples, in comparison to control cultures treated with only DMSO. The percentage of cell viability was calculated as the mean (A570–A630) and compared to cells treated with DMSO alone. The experiments were performed in triplicate. Mean values and standard deviation were calculated on biological triplicates using GraphPad Prism8 software (version 8.0.2) (GraphPad, San Diego, CA, USA).

### 4.6. WST-8 Assay

RPMI 8226 cells were seeded in a 96-well round-bottom microtiter plate at a concentration of 2 × 10^4^ cell/well and incubated at 37 °C and 5% CO_2_ in a humidified cell culture incubator. After 24 h, the medium was replaced with fresh medium containing different concentrations of total extract and fractions dissolved in DMSO (0.5% *v*/*v*). After 72 h of treatment, cell viability was assessed adding the WST-8 assay solution (Abcam, Burlingame, CA, USA) at 1:10 (0.5 mg/mL) in the medium for 4 h at 37 °C and 5% CO_2_. After incubation, the absorbance was measured at OD = 450 nm and 630 nm as background, using a microplate reader (Synergy™ HTX Multi-Mode Microplate Reader, Agilent, Santa Clara, CA, USA). The assay was performed according to the manufacturer’s instructions. Cell viability was expressed as a percentage of viable cells in the presence of the tested samples, in comparison to untreated control cultures with only DMSO. The experiment was performed in triplicate. Mean values and standard deviation were calculated on biological triplicates using GraphPad Prism8 software (GraphPad, San Diego, CA, USA).

### 4.7. Inflammatory Assay

A2058 was seeded in 6-well microtiter plate to a final number of 3.5 × 10^5^ cells/well. After 24 h, the medium was replaced with new medium and cells were treated with 1 µg/mL of LPS and incubated for 1 h, at 37 °C and 5% CO_2_. After the incubation with LPS, cells were treated with 12.5 µg/mL of fraction D of *Halamphora* sp. and incubated for 6 h at 37 °C and 5% CO_2_. The experiment was performed in triplicate. After 6 h of treatment, cells were collected for total RNA extraction and supernatant for ELISA assay. Statistical analysis was performed by GraphPad Prism8 (GraphPad Software Inc., San Diego, CA, USA) by using a two-way analysis of variance (ANOVA) following by Dunnett’s multiple-comparison test (*p*: 0.12 (not significant); 0.033 (*); 0.002 (**); and <0.001 (***)).

### 4.8. Total RNA Extraction, Quality Check and Revers Transcription Quantitative PCR (RT q-PCR) Analysis

Total RNA from A2058 cells was isolated with TRIZOL^®^ reagent (Thermo Fisher Scientific, Waltham, MA, USA) according to manufacturer’s instructions. RNA quantity and quality were evaluated using 1 µL per sample and measuring the absorbance at 260 nm and the 260/280 nm and 260/230 nm ratios using a NanoDrop (ND-1000 UV–Vis spectrophotometer; NanoDrop Technologies, Wilmington, DE, USA). RNA quality was also assessed on 1.5% agarose gel to check that the ribosomal bands were not degraded. Primers sequence, listed in Table 1 for RT-qPCR, were designed using the software Primer3 version 4.1.0 (https://primer3.ut.ee/, accessed on 10 September 2024). The size of amplicons was kept in the range of 150–200 base pairs, the length of primers between 19–20 nucleotides, the melting temperature about 60 °C, and the CG content at about 50%. Then, 1 μg of RNA sample was retrotranscribed into complementary DNA (cDNA) using an iScript^TM^ cDNA Synthesis Kit (BIORAD, Hercules, CA, USA) following the manufacturer’s instructions. A dilution of 1:10 of cDNA was used as a template for RT-qPCR by using a CFX384™ Real-Time System (BioRAD). The PCR volume of each sample was 10 μL with 5 μL of PowerTrack^TM^ SYBR^TM^ Green Master Mix (Thermo Fisher Scientific, Waltham, MA, USA), 0.7 pmol/μL for each oligo, and 1 μL of cDNA as template. To study the expression level of each gene of interest under the various conditions, we used the relative expression software tool (REST- version 2) normalizing to *rplp0* mRNA expression. Statistical analysis was performed by GraphPad Prism8 (GraphPad Software Inc., San Diego, CA, USA) using a two-way analysis of variance (ANOVA) following by Tukey’s multiple-comparison test (*p*: 0.12 (not significant); 0.033 (*); 0.002 (**); and <0.001 (***)).

### 4.9. ELISA Sandwich Assay

A2058 cells were seeded in 96-well microtiter plates at a concentration of 1 × 10^4^ cell/well and after 24 h were treated with 1 ug/mL of LPS for 1 h and then treated with fraction D for 6 h. An enzyme-linked immunosorbent assay (ELISA) was performed immediately to test TNF-*α* secretion. One day prior to ELISA testing for TNF-*α* secretion, EIA/RIA 96-well plates (Corning incorporated, Kennebunk ME, USA) were coated with 2 μg/mL capture antibody (eBioscience, San Diego, CA, USA) and placed at 4 °C overnight. Between each step, plates were washed with tris-buffered saline (TBS; at pH 7.4, with 0.05% Tween-20). All incubations were at room temperature with shaking. A volume of 200 μL blocking buffer (TBS w/2% BSA) was added to the plates and incubated for 1 h. Standard concentrations of TNF-*α* were added to each plate before incubation for 2 h. Biotin-coupled anti-human antibody (eBioscience, San Diego, CA, USA) was diluted in assay diluent (TBS with 1% BSA) to 3 μg/mL and added to each well and incubated for 1 h. Diluted ExtrAvidin^®^–Alkaline Phosphatase (Sigma-Aldrich, St. Louis, MO, USA) was added and plates incubated for 30 min. Then, 100 μL pNPP substrate (Sigma-Aldrich, 1 mg/mL in 1 M diethanolamin buffer pH 9.8) was added to each well and incubated for 45 min. Absorbance was read at 405 nm. Experiments were performed in triplicate. Statistical analysis was performed with GraphPad Prism8 (GraphPad Software Inc., San Diego, CA, USA) using a two-way analysis of variance (ANOVA) following by Tukey’s multiple-comparison test (*p*: 0.12 (not significant); 0.033 (*); 0.002 (**); and <0.001 (***)).

### 4.10. Confocal Immunofluorescence

HaCaT cells were seeded at density of 1 × 10^5^ on glass coverslips in 12-well cell culture plates and kept in a humidified cell culture incubator at 37 °C and 5% CO_2_. After 24 h, cells were treated with 1 µg/mL of LPS and 12.5 µg/mL of fraction D for 6 h. DSMO (0.5%) was used as a control. Cells were fixed and stained following an established protocol [61]. Briefly, they were fixed with 3.6% (*v*/*v*) paraformaldehyde, then permeabilized with 0.25% Triton X-100 and blocked for 30 min with 1% (*w*/*v*) BSA (all diluted in phosphate saline buffer (PBS). The cells were stained with anti-human TNF-*α* (dilution 1:1000, Proteintech^®^, Proteintech, Rosemont, IL, USA, Cat. N: 60291-1-Ig) antibody overnight at room temperature. The following morning, coverslips were washed with PBS (3 × 5 min) and then blocked again in 1% (*w*/*v*) BSA in PBS for 15 min at room temperature. The secondary antibody, Goat anti-Rabbit IgG Alexa Fluor 488 (Invitrogen^TM^, Thermo Fisher Scientific, (Ref. A11034)) was then diluted 1:5000 in 1% (*w*/*v*) BSA in PBS and applied for 1 h at room temperature. The samples were then washed 3 × 5 min in PBS and mounted on glass coverslips using Vectashield DAPI mounting medium (VECTASHIELD Vibrance ^®^ Antifade Mounting Medium with DAPI-2BScientific, 2BScientific, Liberty Corner, NJ, USA)”) and stored at 4 °C until imaged. Coverslips were imaged using an upright confocal microscope using a LEICA SP5 confocal microscope (Leica Microsystems, Wetzlar, Germany), using a 100× objective. A diode laser (405 nm) was used to excite DAPI, and an argon laser was used to excite the Alexa Fluor 488 secondary antibody. Images were taken using the LEICA Application Suite Advanced Fluoresence 2.7.3.9723 software.

## 5. Conclusions

In the current study, we demonstrated that fraction D of *Halamphora* sp. exhibited both antiproliferative and anti-inflammatory effects on human malignant melanoma A2058 cancer cells by regulating the decrease of inflammatory cytokines tumor necrosis factor alpha (TNF-*α*) and interleukin-6 (IL-6) and the chemokine interleukin-8 (IL-8 or CXCL8). Moreover, we also examined gene expression related to apoptosis and migration pathways. To identify the potential molecules responsible for these effects, we conducted a dereplication analysis that revealed hydroxypheophorbide *a* (a chlorophyll degradation product, known to have anti-inflammatory and anticancer properties) as the most abundant compound in fraction D. Given that inflammation plays a major role in tumor progression and immune evasion, anti-inflammatory activity of fraction D could contribute to a more favorable tumor microenvironment, potentially enhancing the effectiveness of existing therapies. Once the compound responsible for the activity observed in vitro has been identified, it could be studied as an adjuvant in immunotherapy. Our studies have been conducted in vitro on human cell lines; therefore, further investigations are necessary to confirm anti-inflammatory activity in vivo to elucidate the mechanism of action involved and evaluate whether these compounds could be taken forward as therapeutics for treatment of melanoma and potentially other cancers.

## Figures and Tables

**Table 1:** List of genes and sequences of primers used for qPCR analysis.

Gene	Accession Number		Primer 5′-3′	Primer 3′-5′
*RPLP0*	NM_001002.4	ribosomal protein lateral stalk subunit P0	TGTGGGAGCAGACAATGTGG	CATTCCCCCGGATATGAGGC
TNF-*α*	NM_000594.4	Tumor necrosis factor α	GACAAGCCTGTAGCCCATGT	AGGTACAGGCCCTCTGATGG
*IL-6*	NM_000600.5	Interleukin-6	TGTGAAAGCAGCAAAGAGGC	TGTACTCATCTGCACAGCTCTG
*STAT3*	NM_003150.4	signal transducer and activator of transcription 3	GGACCCCATTGTACAGCACC	AGGGAATTTGACCAGCAACCT
*CXCL8*	NM_000584.4	C-X-C motif chemokine ligand 8	CTCCAAACCTTTCCACCCCA	TTCCTTGGGGTCCAGACAGA
*JNK1*	NM_002750.5	Mitogen-activated protein kinase 8	AATCTGAGCTTTGACCTCTCGC	CTGAAGCAGAAGCTCCACCA
*JAG1*	NM_000214.3	jagged canonical Notch ligand 1	TCCAACGACACACCTGAAGG	TGTTCCCGTGAAGCCTTTGT
*CSP3*	NC_000019.10	Caspase 3	TGTGGGAGCAGACAATGTGG	TGCTATTGTGAGGCGGTTGT
*BAX*	NM_138761.4	BCL2 associated X, apoptosis regulator	CGACATCTGTACCAGACCGAG	GAGCAGCCCAGAGGCG
*BCL2*	NM_000633.3	BCL2 apoptosis regulator	CGGCGGCAATCATCCTCT	GGTGAACTGGGGGAGGATTG

## Data Availability

The original contributions presented in this study are included in the article/Supplementary Materials. Further inquiries can be directed to the corresponding author.

## Supplementary Materials

The following supporting information can be downloaded at: https://www.mdpi.com/article/10.3390/md24030104/s1, Figure S1: Cell viability assay. The figure shows the effects of *Halamphora* sp. fraction D on cell-viability. MTT assay was used to test the fractions at increasing concentrations (0.1, 0.5, 1, 5, 10, 12.5, 25, 50, 100 μg/mL) on A2058 and HaCaT cell lines for (**a**) 24 h, (**b**) 48 h, (**c**) 72 h. Cell viability was normalized using cells with only DMSO (0.5%) as control sample. Results are expressed as percent survival after 6 h exposure (n = 3; * for *p* < 0.033; ** for *p* < 0.002 and *** for *p* < 0.001, Two-way ANOVA, Dunnett’s test). Figure S2: Real-time PCR. Expression levels of genes involved in inflammatory pathways in LPS-induced HaCaT cells treated with fraction D. The results are expressed in log2 fold-changes. Statistical analyses were performed using two-way ANOVA, Tukey’s test (*** *p*  <  0.001, ** *p*  <  0.01, * *p*  <  0.05). *RPLP0* was used as reference gene.
