# Supplementary material for: Halamphora sp. Reduces Inflammation in LPS-Stimulated Human Malignant Melanoma and Immortalized Keratinocytes Influencing TNF-α Release
Source: Mar Drugs. 2026 Mar 10;24(3):104. doi: 10.3390/md24030104 (PMC13027523; doi:10.3390/md24030104)
Supplement: Supplementary file 1 [file marinedrugs-24-00104-s001.zip › marinedrugs-4175074-supplementary.pdf]

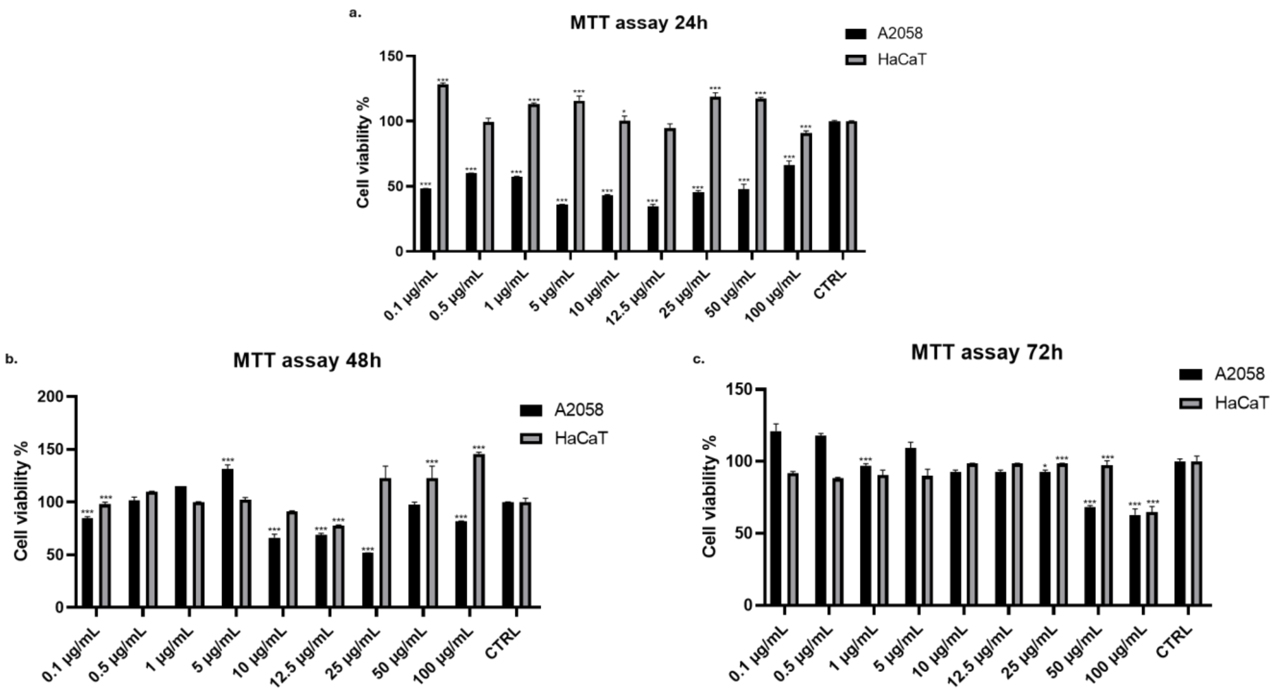

Figure S1. Cell viability assay. The figure shows the effects of *Halamphora* sp. fraction D on cell-viability. MTT assay was used to test at increasing concentrations (0.1, 0.5, 1, 5, 10, 12.5, 25, 50, 100 µg/mL) of fractions on A2058 and HaCaT cell lines for a) 24 h, b) 48 h c) 72 h. Cell viability was normalized using cells with only DMSO (0.5%) as control sample. Results are expressed as percent survival after 6 h exposure (n = 3; *** for p < 0.001, Two-way ANOVA, Dunnett's test)

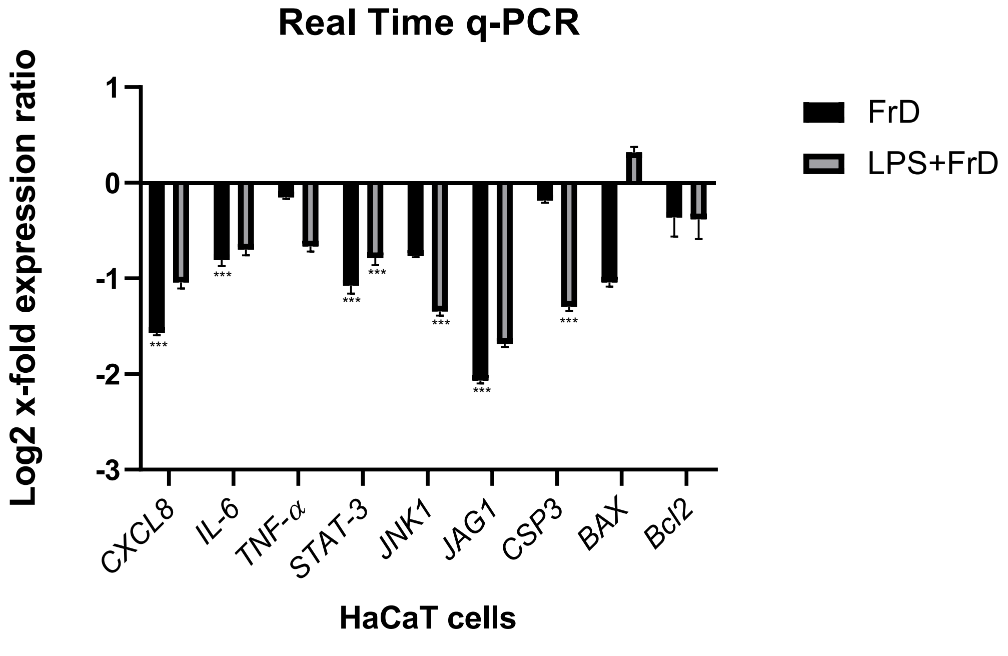

Figure S2. Real Time PCR. Expression levels of genes involved in inflammatory pathways in LPS-induced HaCaT cells treated with fraction D. The results are expressed in log2 fold-changes. Statistical analyses were performed using two-way ANOVA, Turkey's test (*** p < 0.001). RPLP0 was used as reference gene.
